# Anti-saccade as a tool to evaluate cognitive impairment in vestibular migraine

**DOI:** 10.3389/fneur.2024.1419372

**Published:** 2024-06-14

**Authors:** Lingmei Lu, Wenyu Ni, Yin Liu, Li Sun, Fei Li

**Affiliations:** ^1^Department of Neurology, Qidong People’s Hospital/Affiliated Qidong Hospital of Nantong University, Nantong, China; ^2^Department of Endocrinology, Qidong People’s Hospital/Affiliated Qidong Hospital of Nantong University, Nantong, China; ^3^Dizziness Clinic, Jilin Provincial Academy of Chinese Medicine Sciences, Changchun, China; ^4^Department of Neurology, Second Affiliated Hospital of Naval Medical University, Shanghai, China

**Keywords:** vestibular migraine, anti-saccade, cognitive impairment, observational study, evaluation tool

## Abstract

**Background:**

Vestibular migraine (VM), an intricate subtype of migraine, amalgamates the dual attributes of migraine and vestibular disorders. In clinical settings, individuals with VM frequently articulate concerns regarding the manifestation of subjective cognitive impairment. This cognitive dysfunction is intricately linked with diminished mobility, heightened susceptibility to falls, and increased absenteeism in afflicted patients. Consequently, comprehending the features of cognitive impairment in VM patients holds potential clinical significance. The pursuit of rapid and objective methods for detection and assessment is foundational and prerequisite for efficacious cognitive management of VM patients.

**Methods:**

The study encompassed 50 patients diagnosed with vestibular migraine and recruited 50 age-sex matched healthy controls. All participants underwent anti-saccade tasks, and cognitive evaluation was performed using the MMSE and MoCA to assess overall cognitive function. Additionally, RBANS scales were employed to measure specific cognitive domains.

**Results:**

The VM patients and normal controls demonstrated statistical parity in terms of age, gender, education, weight, and BMI, with no significant differences observed. Analysis of cognitive scores divulged a marked increase in the incidence of Mild Cognitive Impairment (MCI) in VM patients compared to Healthy Controls (HCs). Both MMSE and MoCA scores were notably lower in VM patients compared to their healthy counterparts. The RBANS cognitive test indicated significant impairment in immediate memory, visuospatial construction, language, attention, and delayed memory among VM patients. Notably, the Trail Making Test and Stroop Color-Word Test revealed compromised processing speed and executive function cognitive domains. The anti-saccadic task highlighted significantly elevated anti-saccadic latency and frequency of direction errors in vestibular migraine patients. Symptom severity, illness duration, and episode frequency in VM patients positively correlated with counter-scanning errors and negatively correlated with cognitive performance across diverse cognitive domains.

**Conclusion:**

VM patients exhibit cognitive decline across multiple cognitive domains during the interictal period. This cognitive impairment may not be fully reversible, underscoring its potential clinical significance for cognitive management in VM patients. The sensitivity of anti-saccade tasks to the cognitive status of VM patients positions them as promising objective indicators for diagnosis, intervention, and evaluation of cognitive impairment effects in VM in future applications.

## 1 Introduction

Vestibular migraine (VM) constitutes a cluster of disorders characterized by intermittent vestibular symptoms and migraine as its cardinal manifestations. It stands as the second most prevalent cause of vertigo in clinical practice, boasting a lifetime prevalence rate ranging from 1 to 2.7% (1, 2). In 2012, the International Headache Society, in collaboration with the Committee for Classification of Vestibular Disorders of the Bárány Society, jointly formulated diagnostic criteria (3) for VM and probable VM. Subsequently, VM was integrated into the International Classification of Headache Disorders, Third Edition (ICHD-III), formally disseminated in 2018 (4). Since then, VM has garnered widespread acceptance within the vestibular and headache communities, emerging as a focal point of research in the realms of headache and vertigo, both domestically and internationally.

The nexus between vestibular disorders and cognitive impairment has garnered escalating attention in recent years. Studies have revealed that patients with vestibular diseases often exhibit varying degrees of cognitive dysfunction (5, 6). In a cross-sectional analysis of data from the 2008 National Health Interview Survey, Robin T. Bigelow et al. (7) observed an eightfold increase in the likelihood of “serious difficulty concentrating or remembering” among patients with vestibular vertigo in comparison to other adults in the United States. Agrawal et al. (8) reported a heightened prevalence of vestibular dysfunction in individuals with mild cognitive impairment and Alzheimer’s disease, where vestibular dysfunction was three times more prevalent in Alzheimer’s disease patients than age-matched controls (9).

Concurrently, the association between migraine and cognitive impairment is progressively elucidated, with the management of migraine-related cognitive symptoms assuming a pivotal role in migraine episode management post-pain alleviation (10, 11). In clinical scenarios, migraineurs frequently articulate concerns regarding cognitive decline, encompassing information processing speed, basic attention, executive functions, memory, and verbal skills (12). This decline manifests commonly before, during, and after a migraine attack, and treatment during the acute phase does not consistently ameliorate cognitive symptoms. This subjective cognitive decline during attacks finds validation through standardized neuropsychological assessments (13). Recognized as a cause of migraine-related functional disability, cognitive dysfunction during migraine attacks and peri-attacks should be addressed as a valuable secondary endpoint in acute migraine treatment (10). While investigations on cognitive impairment between migraine attacks have not yielded uniform conclusions, a recent meta-analysis indicates that migraineurs exhibit lower general cognitive and language functioning. Moreover, migraine is associated with an elevated risk of all-cause dementia, vascular dementia (VaD), and Alzheimer’s disease (AD) (14).

Vestibular migraine identified as a distinct subtype within the spectrum of migraines, is distinguished by the concurrent manifestation of both migraine and vestibular disorders. Consequently, it is plausible that cognitive impairments, commonly associated with both vestibular disorders and migraine, synergistically contribute to more pronounced cognitive deficits in individuals afflicted with VM. Within clinical settings, a prevalent grievance in VM is the experience of “brain fog”, a subjective description encompassing difficulties in thinking, attention, and memory.

A recent investigation, drawing upon data from the 2016 National Health Interview Survey, disclosed that 52% of VM patients self-reported cognitive dysfunction. This cognitive impairment was found to be correlated with issues related to mobility, an increased risk of falls, and elevated rates of work absenteeism among afflicted individuals (15). However, the precise nature of cognitive impairment in patients with VM has yet to be fully characterized.

In our study, we employed the Mini-Mental State Examination (MMSE) and Montreal Cognitive Assessment (MoCA) to evaluate the overall cognitive function, while the Repeatable Battery for the Assessment of Neuropsychological Status (RBANS) scales were utilized to assess various cognitive domains in both VM patients and control subjects. Additionally, all participants underwent anti-saccade tasks, with the objective of elucidating the specific characteristics of cognitive impairment in VM patients and furnishing referable data to support the clarification of underlying mechanisms governing cognitive impairment in the context of VM.

## 2 Materials and methods

### 2.1 Participants and inclusion/exclusion criteria

**Figure 1:**
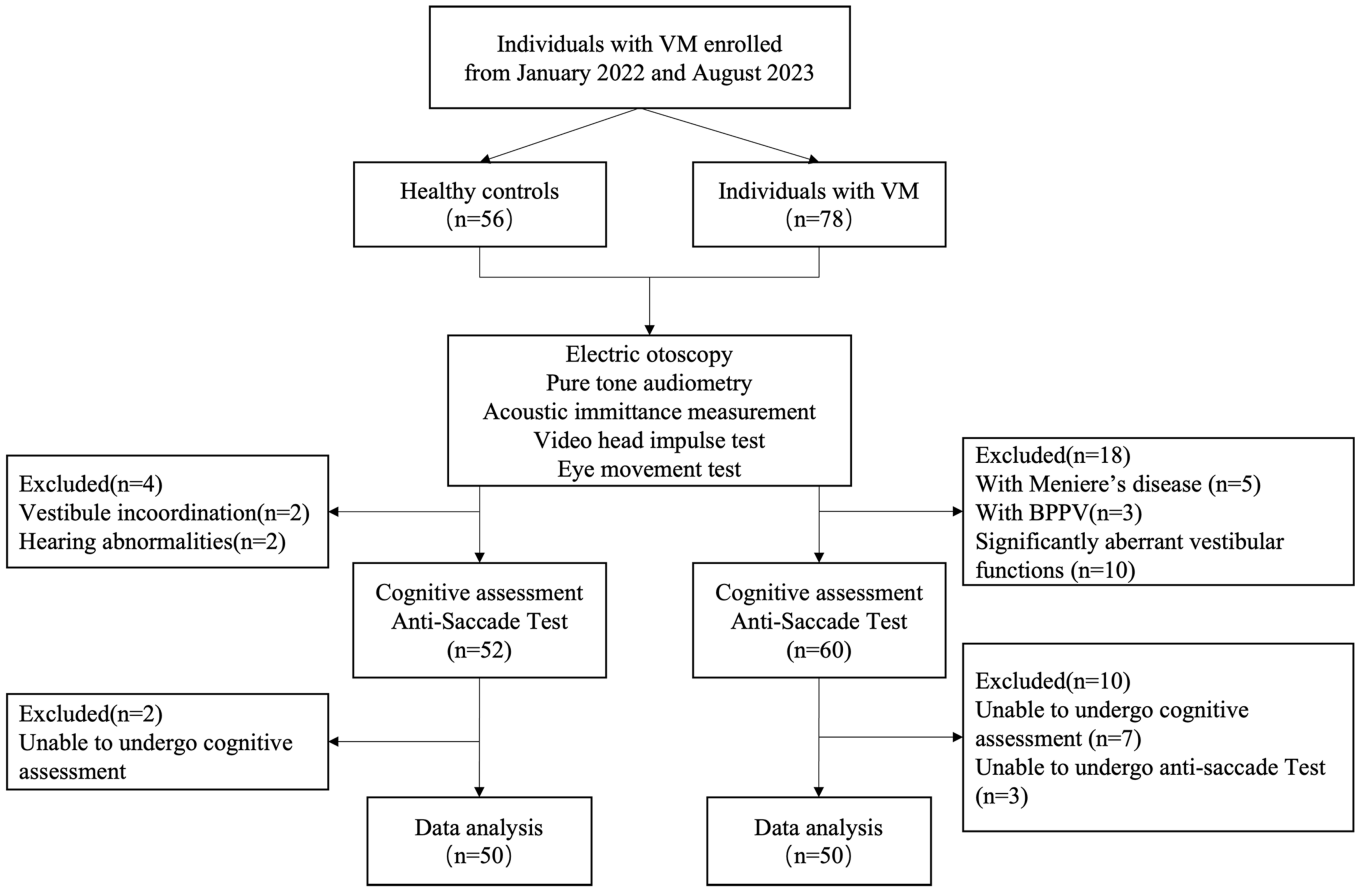
Study sample flow chart.

Between January 2022 and August 2023, we invited consecutive patients presenting with a chief complaint of dizziness, who sought treatment at specialized vertigo clinics in Qidong People’s Hospital, to participate in this study. Additionally, we recruited 50 healthy controls devoid of a history of migraine, dizziness, or any neurological disorders, thereby serving as the normal control group (Figure 1).

The inclusion criteria for the VM group were as follows: (1) Diagnoses of VM were established in adherence to the International Classification of Headache Disorders, 3rd edition^[4]^. Only individuals meeting these criteria were enrolled in the study. (2) Absence of organic neurological or aural diseases. (3) Demonstrating proficient neck movement and oculomotor capabilities. (4) Possessing normal eyesight or corrected vision of 1.0 or above. (5) Attaining normal results in electric otoscopy, pure tone audiometry, acoustic immittance measurement, video head impulse test, and eye movement test. (6) Demonstration of understanding, cooperation with relevant examinations, and a willingness to provide informed consent.

Exclusion criteria encompassed: (1) Presence of dizziness, vertigo, or hearing abnormalities incongruent with VM characteristics. (2) Manifestation of significantly aberrant vestibular functions, evident in abnormalities identified by video head impulse test and eye movement test. (3) Presence of vertical eye deviation, eye diseases, or severe cervical spondylopathy with restricted cervical movement. (4) Inability to comprehend and cooperate during the experiment.

Ethical approval for this study was obtained from the hospital ethics committee at Qidong People’s Hospital, and all participating patients provided informed consent.

### 2.2 General cognition and tests of cognitive domains

The assessment of general cognition utilized the Mini-Mental State Examination (MMSE) and Montreal Cognitive Assessment (MoCA; Beijing Version). A MoCA score falling within the range of 19–25 was indicative of mild cognitive impairment. Additionally, diverse cognitive subdomains were examined using the Repeatable Battery for the Assessment of Neuropsychological Status (RBANS). This included the evaluation of immediate memory (word list and story memory), visuospatial constructional abilities (line orientation and figure copy), language proficiency (picture naming and semantic fluency), attention (digit span and coding), and delayed memory (recognition of word list, story, figure). Low score of each part indicated worse cognitive performance.

To gage processing speed and executive function, the study employed the Trail Making Test (Parts A and B), Stroop Color Word Test (Parts I, II, and III). Trail Making Test is scored by how long it takes to complete the test. It has two parts. Part A consists of 8 circles on a piece of paper, and half of the circles have the numbers 1–4 in them and the other half contain the word one-four. The participants continue to connect the circles in order like this: 1-one-2-two-3-three-4-four. Part B consists of 25 circles on a piece of paper. Similarly, thirteen of the circles have the numbers 1–13 in them and the other twelve contain the word one- twelve. The person is expected to connect the circles in order like this: 1-one-2-two-3-three-4-four-5-five. and so on. Stroop Color Word Test contains three parts. Task 1: Duration to name different color dots; Task 2: Duration to name the colors of neutral words (without color names); Task 3: Duration to read color names that are written in different colors (for instance the word “red” is printed in green ink and the patient is expected to read it as red). Long test duration of above two tests indicated worse cognitive performance.

A meticulously trained examiner administered all tests sequentially, and the entire battery of tests required approximately 60 min to complete, following a predetermined order.

### 2.3 Anti-saccade test

Eye movements were recorded in a dark room using an infrared-illuminated VNG system (VertiGoggles ®, ZT-VNG-II, ZEHNIT Medical Technology Co., Ltd., Shanghai, China) under the VNG system software (VertiPACS®) for the higher oculomotor test module (Tian Integrative Sequence®). The system employed a 640×480 pixel camera at 60 frames per second, utilizing pupil tracking to produce digital eye movement recordings for real-time monitoring and analysis of horizontal and vertical eye movements. A 50-inch monitor with a resolution of 3840×2160 was utilized to display visual targets. Subjects were seated 1.2 meters from the monitor. The visual target appeared as a dark cross within a larger white solid circle on the monitor. Calibration was performed for each participant before the anti-saccade task.

In the anti-saccade task, the trial commenced with the appearance of the central target, which persisted for a randomized duration between 1,000 and 2000 milliseconds. Subsequently, the central target vanished, and a peripheral target emerged in a random direction (either left or right horizontally) and at a random angle (between 10° and 30°) on the monitor, with the target duration being 1,000 milliseconds. The second peripheral target then appeared at the mirror location of the first peripheral target immediately after the first target disappeared. Subjects were instructed to maintain fixation on the central target before any peripheral target appeared, and then to make anti-saccades to the mirror location of the first peripheral target as quickly as possible upon its appearance, continuing to fixate on this location until the second peripheral target appeared. Participants were advised to concentrate on the central target, suppress the natural urge to look at the first peripheral target, and make anti-saccades to the mirror location of the first peripheral target as swiftly as possible upon its emergence, maintaining fixation at this location until the second peripheral target appeared, prompting participants to shift their gaze accordingly. Following this, participants refocussed their gaze to the center of the screen, preparing for the subsequent trial cycle.

The anti-saccade (AS) is defined as the saccadic eye movement to the mirror location of the first peripheral target. The pro-saccade (PS) is defined as the saccadic eye movement to the location of the first peripheral target. Outcome measures for the anti-saccade task include AS error rate (%, the percentage of PS occurrences over total trials), AS latency (ms) and amplitude (degrees), PS latency (ms) and amplitude (degrees), as well as gains for AS and PS. AS error rate was calculated by the percentage of PS occurrences over total trials. AS latency was measured by the duration from the onset of the first peripheral target to the AS. PS latency was measured by the duration from the onset of the first peripheral target to the PS. AS gain is the ratio of AS amplitude to the amplitude of the second peripheral target, and PS gain is the ratio of PS amplitude to the amplitude of the first target. Before the formal test, participants underwent five rehearsals to ensure a thorough understanding of the test procedure and task modalities. Each participant completed 20 formal test trials.

### 2.4 Statistical analyses

Continuous variables are presented as the mean ± standard deviation, and dichotomous variables are expressed as percentages. Independent-samples *t*-tests were employed for comparing continuous variables between two groups, while Pearson chi-square tests were utilized for dichotomous variables. To scrutinize the association between cognitive function, anti-saccade parameters, and clinical symptoms, partial correlation analyses were conducted, with age and educational level as control variables. Statistical significance was considered when *p* was <0.05. The statistical analyses were performed using SPSS v26.0 (IBM, Armonk, NY, USA).

## 3 Results

### 3.1 Clinical characteristics of HC and VM patients

The study subjects were categorized into normal controls and those with VM, with each group comprising 50 cases. Relevant information, including age, gender, education level, body weight, and BMI, underwent analysis. The statistical outcomes indicated no significant differences in age, gender, education, and BMI between the two groups (Table 1).

**Table 1:** Clinical characteristics of HC and vestibular migraine patients.

	HC	VM	*p*
	*n* = 50	*n* = 50	
Age (years)	50.4 ± 10.0	49.66 ± 9.5	0.72
Male sex	22 (44)	20 (40)	0.685
Education (years)	11.4 ± 3.6	10.66 ± 3.27	0.286
Smoking	9 (18)	10 (20)	0.799
Drinking	9 (18)	6 (12)	0.401
Body weight (kg)	66.9 ± 9.2	66.66 ± 8.37	0.874
BMI (kg/m2)	24.2 ± 1.6	24.3 ± 1.1	0.493
SBP (mmHg)	125.5 ± 10.7	127.72 ± 14.5	0.377
DBP (mmHg)	78.4 ± 6.8	81.32 ± 7.85	0.050

Data are presented as mean ± SD or as *n* (%). Comparison of continuous variables between the two groups were analyzed by Independent-samples t-tests, while comparison of categorical variables were analyzed using the *x*^2^ test. SBP, systolic blood pressure; DBP, diastolic blood pressure; BMI, body mass index.

### 3.2 Results of cognitive scores and anti-saccades task in VM patients and HC

**Figure 2:**
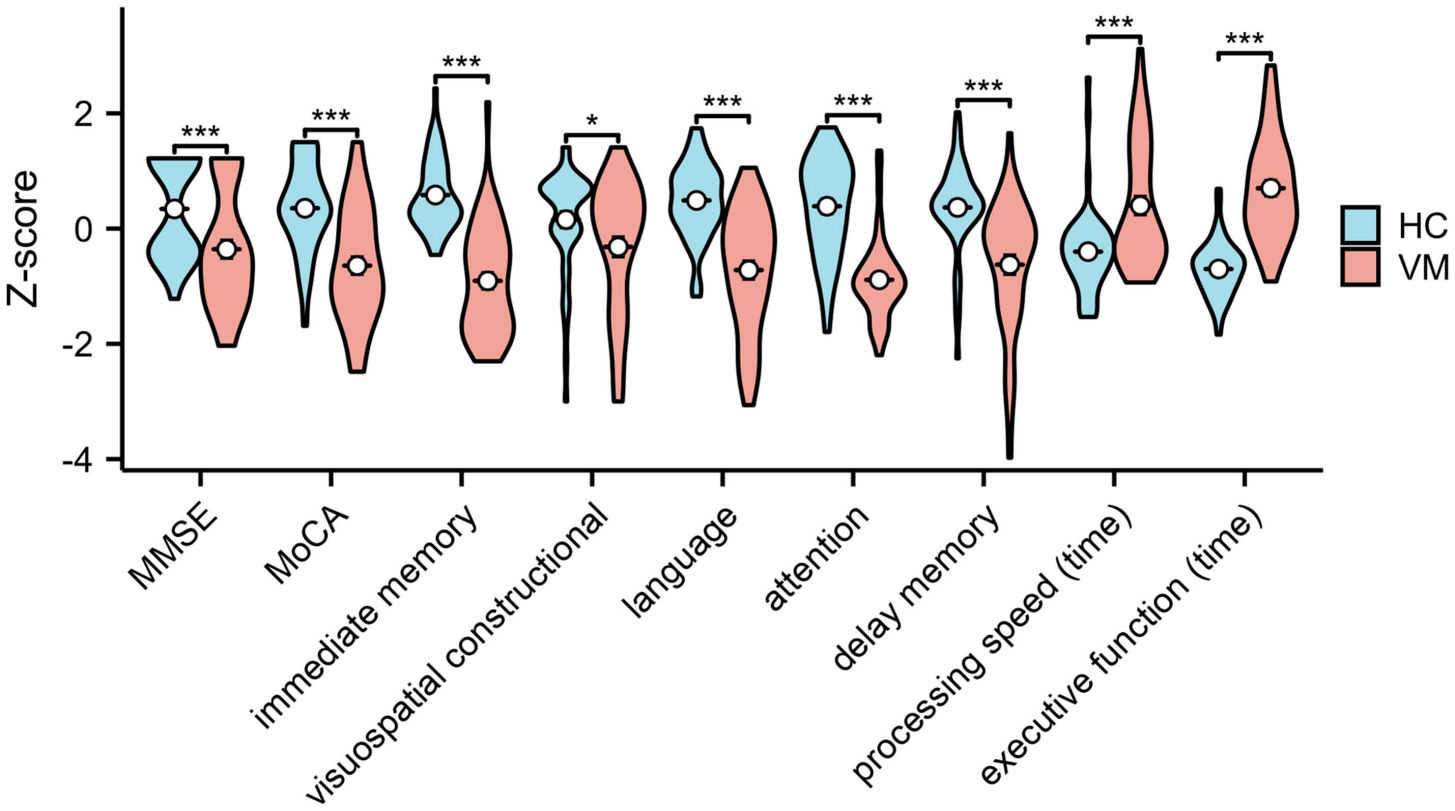
Standard scores of cognitive functions in healthy controls and VM patients. Independent-samples *t*-tests demonstrated significantly decreased cognitive level in VM. *p < 0.05 and ***p < 0.001 was considered significant. MMSE, Mini-Mental State Examination; MoCA, Montreal Cognitive Assessment.

**Figure 3:**
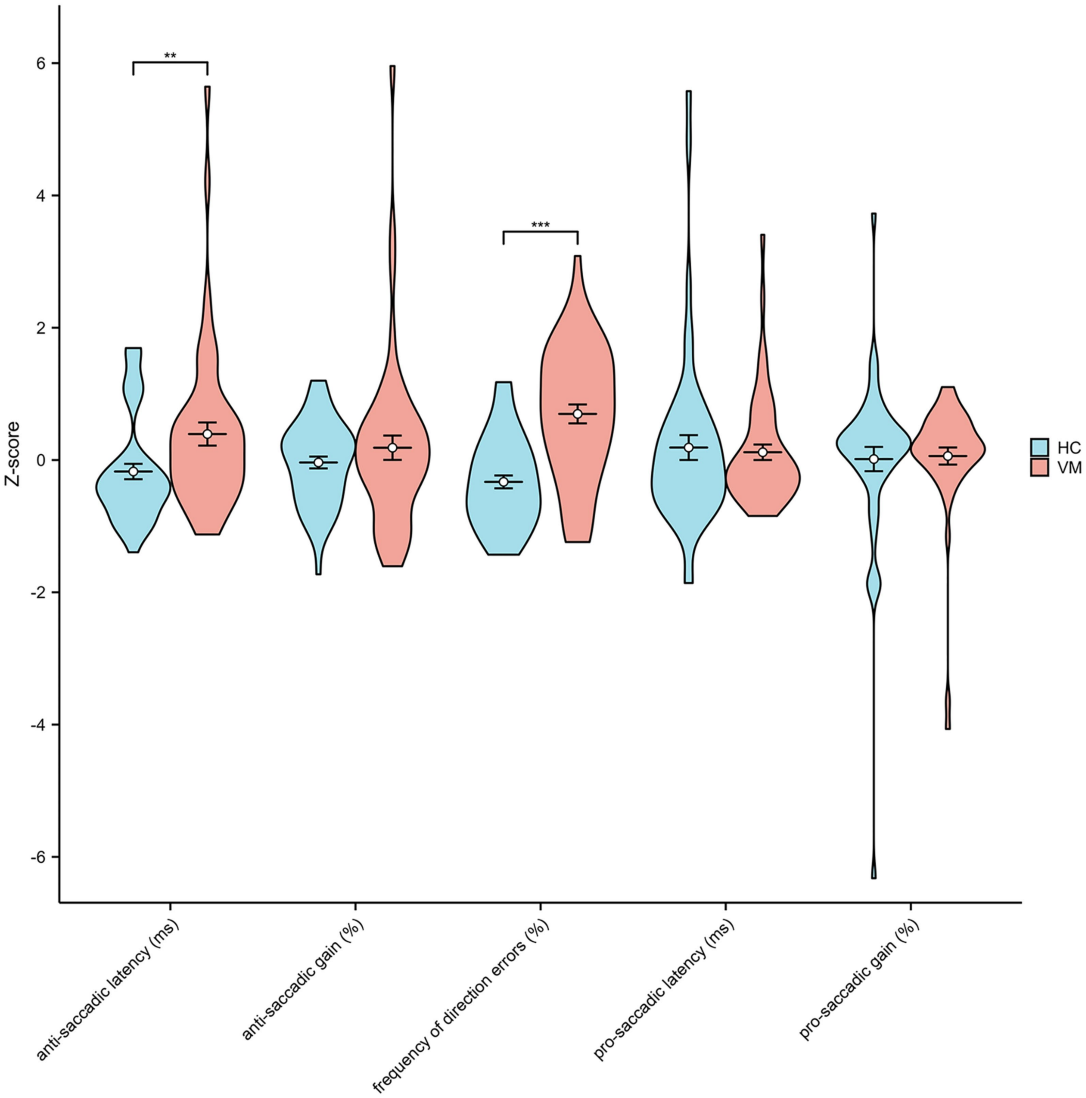
Standard scores of anti-saccade tests in healthy controls and VM patients. Independent-samples *t*-tests showed significantly increased anti-saccadic latency and frequency of direction errors in VM. **p < 0.01 and ***p < 0.001 was considered significant.

Statistical analysis of cognitive scores disclosed a significantly increased incidence of Mild Cognitive Impairment (MCI) in patients with VM compared with Healthy Controls (HCs). The Mini-Mental State Examination (MMSE) scores (28.1 ± 1.3 vs. 28.9 ± 1.1, *p* < 0.01) and Montreal Cognitive Assessment (MoCA) scores (24.6 ± 2.5 vs. 27.1 ± 1.9, *p* < 0.01) were notably lower in VM patients than in healthy controls, indicating an overall decline in cognition in individuals with VM. The Repeatable Battery for the Assessment of Neuropsychological Status (RBANS) cognitive test illustrated a significant impairment in VM patients across immediate memory (70.4 ± 11.7 vs. 88.9 ± 7.6, *p* < 0.01), visuospatial constructional (118.9 ± 8.1 vs. 122.2 ± 6.4, *p* < 0.05), language (93.7 ± 6.1 vs. 100.7 ± 3.8, *p* < 0.01), attention (98.5 ± 10.5 vs. 117.6 ± 12.6, *p* < 0.01), and delayed memory (88.9 ± 12.4 vs. 99.8 ± 8.7, *p* < 0.01) domains. Results from the Trail Making Test (86.8 ± 21.8 vs. 70.3 ± 5.6, *p* < 0.01) and Stroop Color-Word Test (74.5 ± 12.4 vs. 55.0 ± 6.7, *p* < 0.01) indicated impaired processing speed and executive function cognitive domains. Figure 2 demonstrated that standard scores of cognitive functions between two groups. In the domain of eye movement tasks, patients with VM exhibited significantly higher anti-saccade latency (272.2 ± 87.45 vs. 232.2 ± 57.9, *p* < 0.01) and frequency of direction errors (33.46 ± 15.9 vs. 17.3 ± 10.8, *p* < 0.01). The difference in standard scores of anti-saccade tests between HC and VM was significant as shown in Figure 3.

### 3.3 Correlation of anti-saccade parameters with cognitive assessment in all subjects

Upon age and educational level matching, a robust negative correlation was observed between anti-saccade latency, frequency of direction errors, and each cognitive domain, encompassing immediate memory, visuospatial constructional abilities, language proficiency, attention, delayed memory, processing speed, and executive function (Table 2).

**Table 2:** Correlation of anti-saccadic parameters with cognitive assessment in all subjects.

	Anti-saccadic latency		Anti-saccadic gain		Frequency of direction errors	
	*r*	*p*	*r*	*p*	*r*	*p*
MMSE	−0.261	0.010	−0.098	0.338	−0.477	0.000
MoCA	−0.426	0.000	−0.121	0.234	−0.641	0.000
Immediate memory	−0.293	0.003	−0.079	0.44	−0.653	0.000
Visuospatial constructional	−0.278	0.006	0.035	0.731	−0.356	0.000
Language	−0.323	0.001	0.017	0.868	−0.639	0.000
Attention	−0.197	0.051	−0.074	0.467	−0.565	0.000
Delay memory	−0.357	0.000	−0.024	0.817	−0.474	0.000
Processing speed	0.355	0.000	−0.013	0.898	0.570	0.000
Executive function	0.273	0.007	0.111	0.276	0.662	0.000

### 3.4 Correlation between cognitive function, anti-saccade parameters and clinical symptoms in VM patients

Partial correlation analyses conducted among patients with VM revealed that the duration of VM exhibited a negative association with visuospatial constructional abilities and a positive association with the time spent in the processing speed test and the frequency of direction errors. Additionally, both the frequency and duration of headaches displayed negative associations with immediate memory, delayed memory, and language. Furthermore, positive associations were identified between the frequency of direction errors and the duration of VM, frequency of headaches, duration of headaches, and the intensity of pain (Table 3).

**Table 3:** Correlation of clinical symptoms with anti-saccadic parameters and cognitive assessment in VM patients.

	Duration of VM		Frequency of headache		Duration of headache		Intensity of pain		Frequency of vertigo	
	*r*	*p*	*r*	*p*	*r*	*p*	*r*	*p*	*r*	*p*
MMSE	−0.164	0.276	−0.206	0.170	−0.312	0.034	−0.287	0.053	−0.048	0.750
MoCA	−0.102	0.500	−0.262	0.079	−0.259	0.082	−0.142	0.346	−0.104	0.492
Immediate memory	−0.135	0.373	−0.383	0.009	−0.502	0.000	−0.269	0.070	−0.158	0.293
Visuospatial constructional	−0.405	0.005	−0.232	0.120	−0.188	0.212	−0.206	0.170	−0.196	0.191
Language	−0.045	0.765	−0.35	0.017	−0.443	0.002	−0.136	0.366	0.121	0.421
Attention	−0.349	0.017	−0.187	0.214	−0.275	0.065	−0.25	0.093	−0.033	0.828
Delay memory	−0.239	0.109	−0.419	0.004	−0.370	0.011	−0.305	0.039	−0.205	0.172
Processing speed	0.368	0.012	0.284	0.055	0.282	0.058	0.258	0.083	−0.158	0.295
Executive function	0.187	0.215	0.207	0.167	0.312	0.035	0.321	0.030	0.233	0.119
Anti-saccadic latency	0.070	0.642	0.142	0.347	0.072	0.637	0.002	0.988	−0.094	0.534
Anti-saccadic gain	−0.037	0.805	−0.085	0.573	−0.082	0.588	−0.05	0.739	0.261	0.080
Frequency of direction errors	0.345	0.019	0.505	0.000	0.548	0.000	0.365	0.013	−0.072	0.634

## 4 Discussion

The eye movement system serves as an exemplary model for assessing brain function, and eye movement tasks, particularly saccade tasks, have gained widespread usage in recent years to evaluate cognitive function in individuals with neurological and psychiatric disorders (16–18). The anti-saccade test is a method employed to assess the voluntary control of saccades. This task introduces competition between stimulus-induced signals, which tend to elicit automated pro-saccade commands, and voluntary-driven signals, assumed to be goal-directed anti-saccade commands. Correct anti-saccades occur when voluntary signals prevail over automated signals, whereas direction errors manifest when automated signals override voluntary ones (18). The assessment of brain function in the anti-saccade task involves quantitative analysis of “Saccadic Reaction Times” (SRT: the time from stimulus appearance to the first saccade) and the “Anti-saccade Error Rate.” Elevated anti-saccade error rates have been robustly associated with impaired selective attention and executive functioning in studies involving patients with Alzheimer’s Disease (AD) and Mild Cognitive Impairment (MCI) (19–21). Our study, after age and educational level matching, identified a closely negative correlation between anti-saccade latency, frequency of direction errors, and various cognitive domains, including immediate memory, visuospatial constructional abilities, language, attention, delayed memory, processing speed, and executive function. This suggests a potential linkage between anti-saccade performance and general cognition across diverse cognitive domains in both normal individuals and those with vertigo, indicating the potential utility of the anti-saccade test in identifying cognitive decline.

In our investigation, the anti-saccade error rate and saccade reaction time were notably higher in patients with VM than in normal controls during the anti-saccade task. Consequently, we hypothesized varying degrees of cognitive decompensation in individuals with VM. This hypothesis finds validation in our study results, where patients with VM exhibited significantly lower Montreal Cognitive Assessment (MoCA) scores compared to normal controls. Assessments using the Repeatable Battery for the Assessment of Neuropsychological Status (RBANS), Trail Making Test, and Stroop Color-Word Test indicated impairments in multiple cognitive domains among VM patients, including immediate memory, visuospatial abilities, language, attention, delayed memory, processing speed, and executive function. Our findings align with those reported by Wang et al. (22) and Balci B et al. (23) but contradict the results of Demirhan MA et al. (24). The latter study suggests that episodic vestibular disorders like Meniere’s Disease (MD) and VM, characterized by infrequent attacks, do not appear to be associated with cognitive impairment. The discrepancies observed may stem from various factors. Firstly, the study conducted by Demirhan MA et al. (24) featured a relatively small sample size, with only 19 patients for both Meniere’s Disease (MD) and VM, which could potentially diminish the statistical power and hinder the detection of smaller effects. In contrast, our research boasts a larger sample size, with 50 patients for both the VM group and the control group, thereby increasing the likelihood of identifying significant differences. Secondly, there may be differences in patient selection criteria between the two studies, such as the diagnostic criteria for VM, disease duration, and frequency of episodes. These factors could influence the study outcomes. Demirhan MA et al. (24) included VM patients who met the “Barany Society’s International Classification of Vestibular Diseases (ICVD) criteria,” which might encompass both VM and probable VM. In our study, however, patient enrollment strictly adhered to the diagnostic criteria for VM set forth by the International Headache Society (ICHD-3 beta). Additionally, while the study by Demirhan MA et al. (24) did not find significant differences (*p* > 0.05) in baseline matching, the control group’s older age may have introduced a bias in the results. In contrast, our study meticulously matched for not only age and level of education but also gender, weight, and Body Mass Index (BMI). These criteria ensured that the study participants were comparable across key variables, thereby enhancing the accuracy and reliability of our research findings.

Previous studies have indicated a potential association between lower performance in Anti-Saccade Tasks (AST) and structural and functional changes in various frontal regions, including the dorsolateral prefrontal cortex, frontal eye field, and supplementary eye field (25, 26). The frontal cortex governing anti-saccades shares a common physiological structure with memory, language, executive function, and processing speed (27–29). An fMRI study have investigated that migraine patients recruit inhibitory areas, including left frontal pole and orbitofrontal cortex, to accomplish the cognitive task during migraine attacks, a neural signature of their cognitive difficulties (30). Consequently, our hypothesis posits that both cognitive impairment and compromised anti-saccade performance in VM patients may stem from abnormalities in frontal cortex function. The anti-saccade tasks exhibit sensitivity to the cognitive status of VM patients, offering potential as an objective indicator for diagnosing cognitive impairment in VM and evaluating intervention effects in the future.

In conclusion, our study additionally revealed positive correlations between symptom severity, disease duration, and seizure frequency with the anti-saccade error rate. Conversely, negative correlations were observed with cognitive performance across multiple cognitive domains in VM patients. This suggests that cognitive damage induced by VM episodes may not be entirely reversible, underscoring the potential clinical value of cognitive management for patients with VM. Nevertheless, it is noteworthy that the present study did not assess the persistence or dynamic changes of these symptoms over time, representing a limitation that warrants investigation in future studies.

This study is not without its limitations. Initially, the modest sample size can be attributed to our strict adherence to the diagnostic criteria for VM as outlined in the International Classification of Headache Disorders, 3rd edition (beta version). This stringent criterion resulted in the exclusion of a significant number of potential participants. Moreover, to mitigate the confounding effects of organic peripheral vestibular damage on cognitive functions in VM patients, we deliberately excluded those exhibiting substantial organic impairments during the interictal phase. Secondly, while we have diligently excluded the majority of organic pathologies, it is plausible that the cognitive decline observed in patients with VM could be influenced by other unassessed confounding factors, such as sleep disorders and subclinical levels of anxiety and depression. Additionally, although we have meticulously matched the VM group with a control group across various parameters—namely age, gender, educational level, body weight, and BMI—our study did not account for socioeconomic status, a variable that could potentially skew the outcomes. This oversight may introduce a degree of bias into our findings. It is important to consider these limitations when interpreting the results of this study and to acknowledge the areas where further research is warranted to provide a more comprehensive understanding of the subject matter.

## Data availability statement

The raw data supporting the conclusions of this article will be made available by the authors, without undue reservation.

## Ethics statement

The studies involving humans were approved by the hospital ethics committee at Qidong People’s Hospital. The studies were conducted in accordance with the local legislation and institutional requirements. The participants provided their written informed consent to participate in this study. Written informed consent was obtained from the individual(s) for the publication of any potentially identifiable images or data included in this article.

## Author contributions

LL: Funding acquisition, Project administration, Writing – original draft, Writing – review & editing. WN: Writing – original draft, Writing – review & editing, Data curation, Formal analysis, Investigation. YL: Data curation, Writing – review & editing. LS: Data curation, Methodology, Writing – review & editing. FL: Supervision, Writing – original draft, Writing – review & editing, Formal analysis, Methodology.
